# PLGA-Based Co-Delivery Nanoformulations: Overview, Strategies, and Recent Advances

**DOI:** 10.3390/pharmaceutics17121613

**Published:** 2025-12-15

**Authors:** Magdalena M. Stevanović, Kun Qian, Lin Huang, Marija Vukomanović

**Affiliations:** 1 Group for Biomedical Engineering and Nanobiotechnology, Institute of Technical Sciences of SASA, Kneza Mihaila 35/IV, 11000 Belgrade, Serbia; 2 State Key Laboratory for Oncogenes and Related Genes, School of Biomedical Engineering, Institute of Medical Robotics, Med-X Research Institute, Shanghai Jiao Tong University, Shanghai 200030, China; k.qian@sjtu.edu.cn; 3 Department of Clinical Laboratory Medicine, Shanghai Chest Hospital, School of Medicine, Shanghai Jiao Tong University, Shanghai 200030, China; linhuang@shsmu.edu.cn; 4 Shanghai Institute of Thoracic Oncology, Shanghai Chest Hospital, School of Medicine, Shanghai Jiao Tong University, Shanghai 200030, China; 5 Advanced Materials Department, Jozef Stefan Institute, Jamova 39, 1000 Ljubljana, Slovenia; marija.vukomanovic@ijs.si

**Keywords:** nanoparticles, drug release, classification of co-delivery types, fabrication and modification, clinical translation and regulatory considerations

## Abstract

Poly (lactic-co-glycolic acid) (PLGA) is a widely used copolymer with applications across medical, pharmaceutical, and other industrial fields. Its biodegradability and biocompatibility make it one of the most versatile polymers for nanoscale drug delivery. The present review addresses current knowledge and recent advances in PLGA-based co-delivery nanoformulations with a special reference to design strategies, functional mechanisms, and translational potential. Conventional and advanced fabrication methods, the structural design of PLGA-based nanocarriers, approaches to scale-up and reproducibility, classification of co-delivery types, mechanisms governing drug release, surface modification and functionalization are all discussed. Special attention is given to PLGA-based co-delivery systems, encompassing drug–drug, drug–gene, gene–gene and multi-modal combinations, supported by recent studies demonstrating synergistic therapeutic outcomes. The review also examines clinical translation efforts and the regulatory landscape for PLGA-based nanocarriers. Unlike most existing reviews that typically focus either on PLGA fundamentals or on co-delivery approaches in isolation, this article bridges these domains by providing an integrated, comparative analysis of PLGA-based co-delivery systems and elucidating a critical gap in linking design strategies with translational requirements. In addition, by emphasising the relevance of PLGA-based co-delivery for combination therapies, particularly in cancer and other complex diseases, the review highlights the strong clinical and translational potential of these platforms. Key challenges, such as reproducibility, large-scale manufacturing, and complex regulatory pathways, are discussed alongside emerging trends and future perspectives. Taken together, this review positions PLGA-based co-delivery strategies as a critical driver for advancing precision therapeutics and shaping the future landscape of nanomedicine.

## 1. Introduction

### 1.1. Nanocarrier Systems for Drug Delivery

Drug delivery systems play a vital role in maximising the efficacy and safety of pharmaceutical treatments. Traditionally, administering active pharmaceutical ingredients directly has presented challenges with dosing precision, stability, and patient adherence. Controlled drug delivery systems were developed to overcome these limitations by releasing drugs at a controlled rate, improving therapeutic effectiveness while reducing side effects [1,2]. Nanotechnology has transformed pharmaceutical science, especially in the creation of nanoscale drug delivery systems over recent decades [3,4]. These systems, made up of particles typically smaller than 1000 nm, offer key advantages such as increased surface area, improved drug solubility, absorption, and enhanced stability. Nanocarriers can be classified into several categories based on their chemical composition, including polymeric nanoparticles (e.g., poly(lactic-co-glycolic acid) (PLGA)) [5,6], polycaprolactone (PCL) [7], polyethylene glycol [PEG], chitosan, lipid-based carriers (liposomes, solid lipid nanoparticles, nanostructured lipid carriers, ethosomes, transfersomes) [8], inorganic systems (gold, silica, silver, selenium, magnetic nanoparticles, etc.) [9,10,11], and hybrid platforms that integrate multiple material types [12,13]. Each type of carrier brings unique advantages: polymeric systems enable precise control over degradation and drug release, while lipid-based carriers excel in membrane fusion and high drug-loading capacity. On the other hand, unique physicochemical properties of inorganic nanoparticles, including inertness, chemical and thermal stability as well as responsiveness to physical stimuli (light, magnetic field, heat, mechanical force, etc.), relatively simple surface functionalization and specific optical properties provide possibility for external manipulation, bridge delivery of drug with detection, give additional precision to sustained drug release and enable longer shelf-life [14,15,16]. Building on these capabilities, and as nanotechnology in healthcare naturally progresses toward safer and more clinically adaptable materials, biodegradable polymers have, over the past decades, evolved from simple bioresorbable surgical materials into sophisticated platforms for controlled and targeted therapeutic delivery.

### 1.2. Rationale for Using PLGA

Among biodegradable polymers, poly(lactic-co-glycolic acid) (PLGA) stands out for its proven performance and versatility in biomedical applications, particularly as a carrier for controlled drug delivery and as a scaffold for tissue engineering [17]. PLGA is an EMA and FDA-approved copolymer renowned for its mechanical strength, biocompatibility, and capacity to encapsulate a wide range of therapeutic agents, including small molecules, proteins, and nucleic acids such as DNA and RNA. Its widespread adoption stems from a well-established clinical record and a favourable degradation mechanism that supports sustained and predictable drug release, often without the need for surgical intervention. The polymer’s value lies in its unique combination of biodegradability, safety, and tunability. PLGA undergoes hydrolytic degradation in vivo to yield lactic and glycolic acids, both naturally metabolised via the Krebs cycle, thereby avoiding toxic accumulation or chronic immune reactions [18]. Its biocompatibility has been consistently validated through extensive in vitro and in vivo studies, confirming minimal inflammatory and cytotoxic responses when properly formulated [19,20]. In addition, PLGA offers significant design flexibility. By modifying parameters such as the lactide-to-glycolide ratio, molecular weight, and end-group chemistry, researchers can precisely tailor degradation rates and drug release kinetics from days to several months. Compared with other widely used biodegradable polymers such as PCL, PLA, or chitosan, which may suffer from excessively slow degradation (PCL), limited tunability (PLA), or variable physicochemical properties and lower mechanical strength (chitosan), PLGA provides a uniquely balanced combination of controllable degradation and formulation versatility. Combined with its regulatory approval and clinical track record, these features position PLGA as one of the most reliable and adaptable polymers for advanced drug delivery and co-delivery nanoformulations.

### 1.3. Importance of Co-Delivery Strategies

Co-delivery systems have the potential to enhance the therapeutic efficacy of combination therapies by enabling targeted delivery to cells and optimising the pharmacokinetic and physicochemical properties of the therapeutic agents. However, our current understanding of how to effectively tackle complex diseases, such as cancer, remains limited, underscoring the urgent need for systematic investigations to clarify the key factors that govern the performance of co-delivery systems. Defined as the simultaneous delivery of two or more therapeutic modalities, such as drug–drug, drug–gene, or multimodal combinations, co-delivery strategies have emerged as powerful tools to overcome multidrug resistance, achieve synergistic therapeutic effects, and improve overall treatment outcomes. For example, nanoparticle-based platforms that co-encapsulate a cytotoxic agent with a chemosensitizer (a substance that makes tumour cells more sensitive) or small interfering RNA (siRNA) targeting efflux pumps or resistance-related genes can: (i) increase intracellular drug accumulation, (ii) silence resistance pathways at the molecular level, and (iii) provide temporally controlled or sequential release to maximise therapeutic synergy while minimising systemic toxicity. Similarly, co-delivery of a drug and pro-drug is an approach for extending the therapeutic efficacy of the released drug, ensuring the required levels of active drug form and simultaneously providing the form which will be transformed into active form later (after metabolization of the active form), keeping the overall drug’s activity for a longer time period. The approach has particular benefits in the delivery of antibiotics [21,22,23]. Recent reviews and studies document many successful examples and mechanisms [24,25,26,27].

### 1.4. Research Landscape and Methodology of This Review

PLGA-based co-delivery nanoformulations hold significant promise for advancing the treatment and management of multiple chronic conditions, making it essential to stay abreast of recent developments and to systematise findings from diverse studies, thereby enabling meaningful progress in this rapidly evolving field.

Since 2021, an estimated 12,000 research and review papers focusing on PLGA and co-delivery systems have been published, according to Google Scholar. This trend is corroborated by Scopus data, which indicates a steady increase in publications under the keyword “PLGA co-delivery nanoformulation” between 2021 and 2026, with a projected peak in 2025, predominantly within the fields of pharmacology, toxicology, and pharmaceutics. These metrics underscore the accelerating scientific and practical importance of this domain.

The methodology for this review was based on a systematic analysis of peer-reviewed literature sourced from major scientific databases, including PubMed, Scopus, and Web of Science. The search strategy employed combined keywords such as “PLGA,” “drug delivery,” “co-delivery,” “nanoparticles,” “nanoformulations,” “synthesis,” “surface functionalization,” “physicochemical characterisation,” “biological interactions,” “safety assessment,” “clinical translation,” and “regulatory consideration.”

Studies were eligible for inclusion based on their relevance to PLGA-based co-delivery nanoformulation design, nanomaterial integration, demonstrated or potential applications in controlled and sustained therapeutic delivery. Both experimental and review articles were included, with emphasis placed on publications from the last five years to capture the latest advancements. Exclusion criteria primarily encompassed non-PLGA systems, purely theoretical studies without experimental or methodological relevance, conference abstracts, theses, patents, non-peer-reviewed material, and publications lacking sufficient methodological detail. In addition, given the growing attention this topic has received and the surge of publications over the past 5 years, the primary focus has been on PLGA-based co-delivery systems discussed in the literature, which primarily involve parenteral applications. The curated literature was critically evaluated and thematically structured across key sections addressing synthesis approaches, functionalization strategies, co-delivery mechanisms, and application-specific insights. This framework ensures a comprehensive overview of current developments while identifying persisting knowledge gaps and emerging opportunities in the field.

## 2. PLGA: Chemical Structure and Physicochemical Properties

### 2.1. Chemical Structure and Physicochemical Parameters

**Figure 1:**
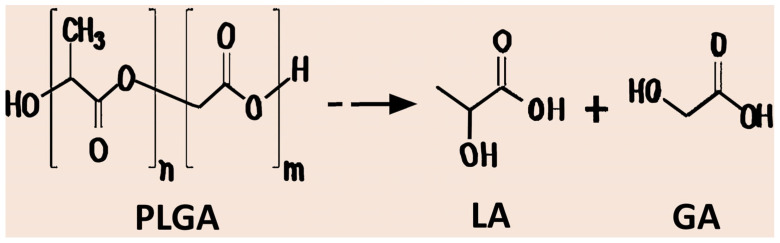
Chemical structure of PLGA polymer. PLGA undergoes hydrolytic degradation into its constituent monomers, lactic acid (LA) and glycolic acid (GA).

PLGA is a copolymer made from poly(lactic acid) (PLA) and poly(glycolic acid) (PGA) units, linked via ester bonds in a linear chain, with properties determined by the ratio of lactide to glycolide, molecular weight, and terminal functional groups [28]. The lactic: glycolic ratio significantly affects hydrophobicity, crystallinity, and degradation kinetics. Higher lactide content increases hydrophobicity and slows hydrolytic degradation, whereas higher glycolide content accelerates water uptake and polymer breakdown, except for a 50:50 ratio of PLA/PGA, which exhibits the fastest degradation [5]. Molecular weight governs mechanical strength, viscosity, and release behaviour, with higher molecular weight PLGA exhibiting slower degradation and more sustained drug release [29]. PLGA crystallinity and melting point depend on multiple factors, including molecular weight, the proportion of lactic and glycolic units, and the regularity of the polymer chain. The end-group chemistry, typically acid-terminated or ester-terminated, further modulates polymer degradation and drug release profiles, where acid-terminated PLGA generally degrades faster due to increased hydrophilicity and autocatalytic hydrolysis, potentially affecting drug stability, whereas ester-terminated PLGA shows slower breakdown [30]. PLGA degradation products, lactic (LA) and glycolic acids (GA), are naturally metabolised via the Krebs cycle, ensuring excellent biocompatibility and minimal systemic toxicity, which supports its widespread use in FDA-approved drug delivery systems [31]. Figure 1 shows the chemical structure of PLGA polymer. So, the physicochemical properties of PLGA, such as lactide-to-glycolide ratio, molecular weight, and end-group chemistry, critically influence degradation rate, drug release kinetics, and mechanical performance. A thorough consideration of these parameters is essential for the rational design of PLGA nanoparticles with controlled and adjustable drug delivery profiles.

### 2.2. Applied Properties and Limitations

Recognised for its biodegradability, biocompatibility, and FDA-approved clinical use [32] PLGA serves as a versatile nanocarrier with degradation rates that can be tuned, as already mentioned above, via the lactide: glycolide ratio, molecular weight, and end-group chemistry, allowing controlled drug release from immediate to prolonged delivery [33]. PLGA nanoparticles can encapsulate a broad range of therapeutic agents, including small molecules, peptides, and nucleic acids, and protect them from enzymatic degradation, improving bioavailability and pharmacokinetics [34]. Moreover, PLGA surfaces can be functionalized with targeting ligands, polyethylene glycol, or other polymers to enhance tissue specificity, circulation time, and reduce immunogenicity [35]. Despite these advantages, PLGA nanocarriers have some limitations. Hydrolytic degradation produces acidic byproducts, which can destabilise pH-sensitive drugs and trigger local inflammation at high doses [36]. Burst release during the initial phase can result in suboptimal dosing, while encapsulation efficiency for highly hydrophilic molecules remains challenging [37]. Additionally, PLGA nanoparticles often require complex fabrication methods and stabilisers, increasing production cost and limiting large-scale reproducibility. Particle stability and preventing aggregation under physiological conditions and ensuring longer and more reliable circulation in blood stream and more effective crossing through different biological barriers require the application of surface-active substances. Their selection must be very careful to avoid toxicity and activation of the immune response [38]. Understanding these strengths and limitations is critical for designing effective PLGA-based drug delivery systems.

## 3. Fabrication Strategies for PLGA-Based Nanoformulations

### 3.1. Conventional Techniques for PLGA Nanoparticle Fabrication

PLGA nanoparticles are commonly prepared using emulsion-based and precipitation methods, which offer reliable control over particle size, encapsulation efficiency, and drug release profiles. The single emulsion–solvent evaporation technique is widely used for encapsulating hydrophobic drugs, involving the emulsification of a PLGA–drug solution in an aqueous phase followed by solvent removal to form solid nanoparticles [39]. For hydrophilic molecules, double emulsion–solvent evaporation (water-in-oil-in-water, W/O/W) is preferred, where the drug is first emulsified into the polymer solution before creating a secondary emulsion, ensuring higher encapsulation efficiency and reduced leakage [40]. Nanoprecipitation (solvent displacement) is another straightforward method, where rapid mixing of a PLGA solution in a water-miscible organic solvent with an aqueous phase induces nanoparticle formation through supersaturation and polymer precipitation; this method is particularly suitable for small hydrophobic drugs and allows for narrow size distribution [41]. Spray drying provides a scalable approach for producing dry PLGA nanoparticles or microparticles, transforming emulsions or polymer solutions into powders while retaining bioactivity, and is increasingly applied for pulmonary or oral delivery [42]. Additional mechanical homogenization, including application of high-velocity homogenisers, ultrasonic dispersing and high-intensity sonochemical de-agglomerations, as well as controlled drying procedures, like freeze drying, are commonly coupled with these techniques [43,44].

Regardless of the method used to produce the particles, one of the key aspects is controlling the size of the resulting nanoparticles, which is most commonly influenced by parameters such as the agitation rate, solvent type or surfactant concentration [45,46,47]. Agitation rate during emulsification directly affects droplet size: higher stirring or sonication speeds produce smaller, more uniform nanoparticles, whereas low agitation leads to larger, polydisperse particles. Solvent type affects polymer solubility and interfacial tension; water-miscible solvents like acetone or acetonitrile generally yield smaller nanoparticles during nanoprecipitation, while less miscible solvents produce larger particles. Surfactant concentration, such as polyvinyl alcohol (PVA), stabilises emulsion droplets; higher concentrations reduce coalescence and particle size, whereas insufficient surfactant can result in aggregation and broader size distributions. Comparative studies indicate that agitation rate and surfactant concentration exert the strongest influence on particle size, while solvent type primarily modulates nucleation and initial particle formation.

Each technique offers distinct advantages and limitations regarding particle size control, drug encapsulation efficiency, and scalability, which must be carefully matched to the intended therapeutic application.

### 3.2. Advanced Techniques for PLGA Nanoparticle Fabrication

Recent advances in PLGA nanoparticle production leverage microfluidics, supercritical fluid processing, electrospraying, and 3D printing to improve reproducibility, scalability, and functional performance. PLGA microfluidic nanoparticle production commonly employs flow-focusing or co-flow microdevice configurations [48]. These designs provide precise control over fluid flow and mixing, allowing the generation of nanoparticles with narrow size distributions, high loading efficiency, and adjustable release characteristics [49]. Supercritical fluid technology, such as supercritical CO_2_, allows solvent-free or low-solvent nanoparticle formation, minimising residual solvent toxicity while producing uniform particles with high surface area, advantageous for poorly soluble drugs [50]. Electrospraying involves applying an electric field to a polymer solution to form fine droplets that solidify into nanoparticles, offering excellent control over particle morphology, size, and surface characteristics, which is beneficial for targeted or stimuli-responsive delivery [51]. Finally, 3D printing has emerged as a promising approach for creating PLGA-based scaffolds or drug-loaded matrices with customizable geometry, porosity, and multi-drug loading, enabling localised and sustained release for tissue engineering and combination therapy applications [52]. However, thermal processes in some 3D printing methods may induce partial PLGA degradation, potentially affecting polymer molecular weight, mechanical properties, and drug release profiles. These factors must be carefully considered during design and fabrication. Collectively, these advanced techniques extend the capabilities of conventional methods, offering precise control, reproducibility, and novel functionalities suited to complex therapeutic needs. Table 1 summarises commonly used methods for PLGA preparation, providing a brief overview of each technique along with its key advantages and limitations.

### 3.3. Optimisation Parameters for PLGA Nanocarriers

The performance of PLGA-based nanocarriers is highly dependent on critical optimisation parameters, including particle size, encapsulation efficiency, and stability. Particle size directly influences crossing biological barriers and biodistribution, cellular uptake, and clearance rates; smaller nanoparticles (<200 nm) typically enhance tissue penetration and circulation time, while larger particles may provide sustained local release [60]. Encapsulation efficiency determines the therapeutic payload delivered per particle and is affected by drug-polymer interactions, solvent selection, and fabrication technique; high encapsulation ensures effective dosing and minimises premature release [61]. Stability, both physical (aggregation, sedimentation) and chemical (polymer degradation, drug leakage), is crucial for storage, handling, and in vivo performance [62]. Surfactants such as polyvinyl alcohol (PVA), poloxamers, and polyethylene glycol (PEG) are commonly employed to stabilise PLGA nanoparticles by reducing interfacial tension and preventing aggregation. PVA is widely used for its strong emulsifying properties and ability to produce uniform particles, poloxamers enhance steric stabilisation and prolong circulation, while PEGylation improves colloidal stability and reduces opsonisation [63]. Moreover, surfactant type and concentration directly influence particle size and encapsulation efficiency, as optimal stabilisation prevents coalescence and drug leakage during fabrication. Other factors, including lyophilisation protocols and polymer molecular weight, are also optimised to maintain long-term stability. Systematic optimisation of these parameters is essential to achieve reliable, reproducible, and clinically relevant PLGA nanocarriers for diverse therapeutic applications [64].

### 3.4. Structural Designs: Single-Carrier Encapsulation, Core–Shell, Layered, or Hybrid Lipid–Polymer Architectures

**Figure 2:**
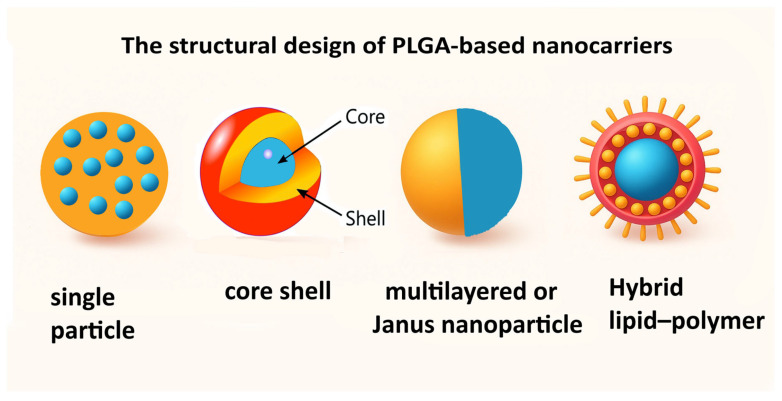
The structural design of PLGA-based nanocarriers: single-carrier encapsulation, core–shell, multilayered or Janus nanoparticle and hybrid lipid–polymer architectures.

The structural design of PLGA-based nanocarriers critically influences drug loading, release kinetics, and therapeutic performance (Figure 2). Single-carrier encapsulation represents the simplest approach, where one or more payloads are uniformly distributed within the polymer matrix. This design is typically prepared using emulsion–solvent evaporation or nanoprecipitation methods, offering straightforward fabrication and controlled release, though it may lack precise spatial segregation for multiple agents [65]. Core–shell architectures separate distinct functional components, such as hydrophilic and hydrophobic drugs or drug and gene payloads, allowing sequential or stimuli-responsive release [66]. Core–shell nanoparticles can be fabricated using a variety of strategies, including two-stage seeded emulsion polymerization, suspension crosslinking, coacervation, interfacial polymerization, solvent evaporation, and vesicle polymerization [47]. In addition, hybrid approaches that combine mini- and microemulsion techniques with these methods have been employed to precisely engineer polymeric nanocontainers and nanocapsules with tunable core–shell structures. These approaches allow independent control over the core and shell compositions, enabling the simultaneous encapsulation of hydrophilic and hydrophobic payloads or the co-delivery of drugs and genetic material. Core–shell systems are particularly advantageous for co-delivering two drugs with differing solubility or release profiles, enabling delayed and prolonged activity [22]. Layered designs, including multilayered or Janus nanoparticles, integrate chemically distinct components within a single structure, providing spatial segregation and independent release profiles [67]. These are often produced via sequential polymer deposition or microfluidic-assisted assembly, offering enhanced stability and multifunctionality. Hybrid lipid–polymer systems combine the structural integrity of PLGA with the biocompatibility and membrane-mimicking properties of lipids. Typically prepared by coating PLGA cores with lipid bilayers or incorporating lipid-polymer blends, these systems improve cellular uptake, endosomal escape, and circulation time [68]. These diverse architectures allow fine-tuning of therapeutic delivery, targeting efficiency, and multifunctionality, making them highly adaptable for combination therapies and complex biomedical applications. Specific design choices should be guided by payload properties, release requirements, and intended clinical outcomes.

### 3.5. Approaches to Scale-Up and Reproducibility of PLGA Nanocarriers

Translating PLGA-based nanocarriers from laboratory to clinical or industrial production requires careful optimisation of scale-up processes to ensure reproducibility, regulatory compliance, and therapeutic efficacy. Maintaining consistent particle size, drug loading, and morphology across batches is critical for regulatory approval and therapeutic efficacy. Microfluidic platforms and continuous-flow reactors have emerged as promising strategies, providing precise control over mixing, solvent diffusion, and nucleation processes, which reduces batch-to-batch variability [69]. Process parameters, such as polymer concentration, flow rates, solvent selection, pH and temperature, must be rigorously standardised to ensure reproducibility during scale-up. Solvent choice is particularly important since many PLGA-compatible organic solvents are environmentally hazardous or incompatible with regulatory guidelines, necessitating careful solvent selection or replacement with greener alternatives. Drying methods such as freeze-drying or spray-drying are essential for long-term stability but pose economic and energy challenges at an industrial scale. Furthermore, quality-by-design (QbD) approaches and statistical design of experiments (DoE) are increasingly employed to identify critical process parameters and optimise production for large-scale manufacturing while maintaining regulatory compliance [70]. For instance, DoE-based optimisation has been shown to minimise size distribution variability and maximise encapsulation efficiency for drug-loaded PLGA nanoparticles at scale. Collectively, these strategies facilitate the industrial and clinical translation of PLGA nanocarriers, providing formulations with predictable performance, enhanced safety, and consistent therapeutic outcomes.

## 4. PLGA-Based Co-Delivery Systems

### 4.1. Classification of Co-Delivery Types

**Figure 3:**
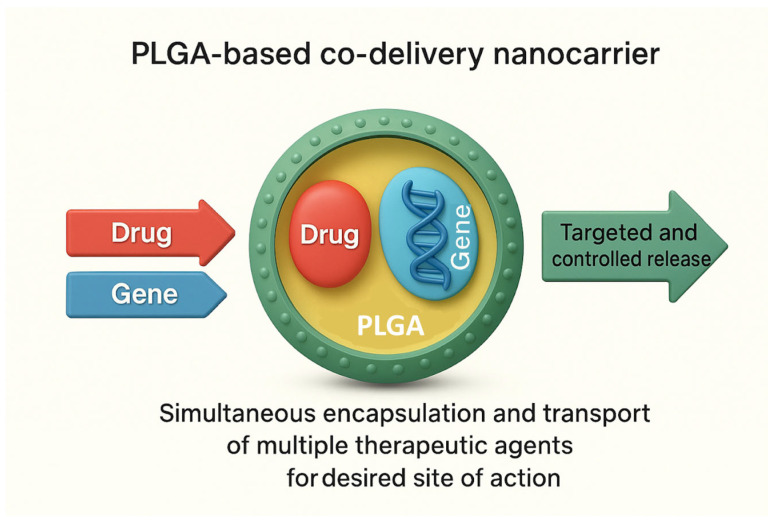
Schematic illustration of a PLGA-based co-delivery nanocarrier for combined drug and gene delivery.

Co-delivery strategies using PLGA-based nanocarriers can be broadly classified based on the nature and combination of the therapeutic cargos. The most common types include dual-drug delivery, where two small-molecule drugs are encapsulated to achieve synergistic therapeutic effects or overcome multidrug resistance [71]; drug–gene delivery, in which a chemotherapeutic agent is co-encapsulated with nucleic acids such as siRNA, miRNA, or plasmid DNA to modulate gene expression and enhance treatment efficacy [72]; gene–gene co-delivery which employ nanocarriers to transport multiple genetic payloads simultaneously, and multi-modal delivery, where drugs are combined with imaging agents, immunomodulators, or photothermal/photodynamic agents to enable theranostic or multi-functional therapeutic approaches [32]. These classifications help in rationally designing nanocarriers according to the therapeutic objective, whether it is synergistic cytotoxicity in cancer, targeted gene silencing, or combining therapy with diagnostic capabilities. Each type poses distinct challenges in terms of loading efficiency, release kinetics, and stability, which must be carefully optimised for effective co-delivery. Figure 3 represents a schematic illustration of a PLGA-based co-delivery nanocarrier depicting the simultaneous encapsulation and transport of multiple therapeutic agents (e.g., drug and gene) for targeted and controlled release at the desired site of action.

### 4.2. Drug–Drug Co-Delivery

Drug–drug co-delivery using PLGA-based nanocarriers enables the simultaneous administration of two or more therapeutic agents, often with complementary mechanisms of action, to achieve synergistic effects and improved treatment outcomes. In oncology, this approach allows combination chemotherapy to overcome multidrug resistance and enhance tumour cell cytotoxicity by co-encapsulating drugs such as doxorubicin and paclitaxel, or cisplatin and curcumin, within the same nanoparticle [73]. Synergistic effects in PLGA-based drug–drug co-delivery systems arise when combined agents activate complementary therapeutic pathways. In cancer therapy, this may involve enhanced apoptosis, inhibition of survival signalling, or sensitisation of tumour cells to treatment, which helps reduce multidrug resistance, for example, through the regulation of pro-apoptotic factors and suppression of resistance-related pathways. An example of such a system is doxorubicin–curcumin PLGA NPs prepared by the solvent evaporation method for the treatment of multidrug-resistant oesophageal carcinoma [74]. Therefore, targeting apoptosis could initiate programmed cell death of cancer cells and improve their response to anticancer drugs. In inflammatory disorders, dual-drug PLGA formulations can co-deliver anti-inflammatory agents and antioxidants to simultaneously reduce oxidative stress and suppress inflammation, thereby accelerating tissue repair [75]. In anti-inflammatory applications, synergy can result from simultaneously targeting pathways like NF-κB and NLRP3 signalling, resulting in reduced inflammation and tissue damage, as shown for curcumin–bioperine PLGA nanoparticles [76]. In the delivery of antibiotics, the formulations which include pro-drug and drug forms (i.e., clindamicin-2-phosphate and clindamycin base) are used to ensure overall drug activity in a prolonged period of time and prevent conditions for bacterial resistance [22]. Sustained release from PLGA carriers helps maintain bactericidal levels and limits bacterial adaptation. Overall, synergy reflects coordinated pharmacodynamics combined with the controlled, co-localised delivery enabled by PLGA nanocarriers [32]. Drug–drug co-delivery also allows controlled and synchronised release profiles, reducing systemic toxicity and minimising adverse effects compared to free drug combinations. This strategy provides a versatile platform to rationally design combination therapies for diverse clinical applications, with a focus on maximising efficacy and patient safety.

However, in the context of drug–drug systems, as well as the additional systems discussed below, it is important to note that chemical interactions between co-encapsulated therapeutic cargos and the PLGA matrix can significantly influence formulation stability, release behaviour, and overall therapeutic performance. Depending on their physicochemical properties, two drugs may interact through hydrogen bonding, ionic interactions, π–π stacking, or hydrophobic association within the polymeric network [77]. Such interactions can modify drug distribution within the nanoparticle core, potentially altering encapsulation efficiency or leading to competitive binding sites within PLGA. In some cases, acidic degradation products of PLGA (e.g., lactic and glycolic acid) may catalyse hydrolysis or promote the chemical instability of sensitive drugs, an effect that can be intensified when one co-loaded drug modifies the microenvironmental pH. Conversely, co-encapsulation can sometimes enhance stability, for instance, a hydrophobic agent may shield a labile hydrophilic drug from premature degradation. These intermolecular and polymer–drug interactions underscore the need for careful pre-formulation studies, including solid-state characterisation, compatibility assessment, and monitoring of drug integrity during PLGA degradation, to ensure optimal stability and performance of drug–drug co-delivery systems.

### 4.3. Drug–Gene Co-Delivery

Drug–gene co-delivery systems leverage PLGA-based nanoparticles to simultaneously transport small-molecule drugs and genetic material, such as DNA, siRNA, or mRNA, enabling synergistic therapeutic effects and targeted modulation of cellular pathways. This approach is particularly valuable in cancer therapy, where co-delivery of chemotherapeutics with siRNA targeting drug-resistance genes can sensitise tumour cells and enhance cytotoxicity. PLGA nanoparticles protect nucleic acids from enzymatic degradation, improve cellular uptake, and allow spatiotemporally controlled release of both drug and gene cargo, optimising therapeutic windows [78]. Recent studies have demonstrated successful co-delivery of doxorubicin with siRNA or mRNA for inducing apoptosis and immune modulation in tumour models, highlighting the versatility of these systems for precision medicine applications [79].

Although PLGA efficiently encapsulates small-molecule drugs such as doxorubicin, its inherent negative surface charge and limited affinity for nucleic acids often necessitate polymer modification to stably load siRNA, mRNA, or DNA. To enable electrostatic complexation with nucleic acids, PLGA nanoparticles are commonly functionalized with cationic polymers or lipids, for example, polyethyleneimine (PEI), chitosan, or poly-L-lysine, and lipid components like DOTAP are frequently employed to introduce a positive charge and condense genetic cargo [80,81]. These cationic additives not only improve nucleic acid encapsulation efficiency but also facilitate endosomal escape by mechanisms such as the proton-sponge effect or membrane destabilisation, thereby boosting intracellular delivery of siRNA [82]. Alternative design strategies include the synthesis of PLGA–PEG–amine block copolymers, surface decoration with cell-penetrating peptides, or using layer-by-layer (LbL) assembly to add sequential oppositely charged coatings around a PLGA core [83]. Such chemical and structural modifications have proven essential for co-encapsulating doxorubicin and siRNA in a stable, bioactive form, enabling coordinated release and effective gene silencing in target cells [84,85]. Furthermore, PLGA-based co-delivery nanoparticles offer the potential to reduce systemic toxicity and overcome the limitations of monotherapy by achieving combined pharmacological and genetic intervention in a single platform.

### 4.4. Gene–Gene Co-Delivery

Gene–gene co-delivery systems utilise PLGA nanoparticles to simultaneously transport multiple genetic payloads, such as CRISPR/Cas components, dual siRNAs, or plasmid DNA constructs, to achieve precise and synergistic modulation of cellular pathways [86]. This strategy enables combinatorial gene editing or knockdown, which is particularly useful in cancer, metabolic disorders, and genetic diseases, where targeting multiple genes can overcome compensatory mechanisms and enhance therapeutic efficacy. PLGA-based carriers protect nucleic acids from enzymatic degradation, improve intracellular delivery, and allow for controlled release kinetics, ensuring synchronised gene activity. Recent advances have demonstrated the successful co-delivery of sgRNA for targeted genome editing, as well as dual siRNA systems to silence multiple oncogenes, resulting in enhanced efficacy and reduced off-target effects. These multifunctional gene–gene co-delivery platforms underscore the potential of PLGA nanoparticles in next-generation genetic therapies, offering tunable dosage, spatial control, and minimised systemic toxicity.

Co-encapsulation of genetic payloads with vastly different sizes and polarities, such as CRISPR/Cas ribonucleoprotein complexes, siRNAs, or large plasmid DNA constructs, poses significant formulation challenges for PLGA-based carriers [87,88,89,90]. Small, highly anionic siRNAs readily form compact polyplexes with cationic surface modifiers, whereas large plasmids and bulky CRISPR/Cas RNPs require more spacious compartments and stronger stabilising interactions to prevent aggregation or degradation. Differences in hydrodynamic size, charge density, and rigidity can lead to uneven distribution within the PLGA matrix, reduced encapsulation efficiency, or premature leakage of smaller cargo. Moreover, PLGA’s hydrophobic core favours incorporation of small hydrophobic molecules, while hydrophilic macromolecules must rely on electrostatic or ionic interactions for stable loading, creating potential competition between payloads. These constraints often necessitate advanced formulation strategies, such as pre-complexation of nucleic acids with cationic polymers, use of layered or core–shell architectures, microfluidic-assisted fabrication, or modular assembly of multiple compartments to maintain structural integrity and ensure balanced co-loading. Addressing these size- and polarity-related challenges is critical for achieving synchronised release profiles and maintaining the biological activity of each component in gene–gene co-delivery systems.

### 4.5. Multi-Modal Co-Delivery

In addition to simpler dual cargo systems, PLGA have increasingly been engineered for multi-modal/theranostic applications, where drugs are co-delivered with, for example, imaging agents, immunomodulators, or photothermal/photodynamic agents [32]. These multi-delivery platforms enable more complex, multifunctional therapeutic strategies such as real-time imaging, controlled release, and synergistic therapy (e.g., chemo-photothermal, immuno-phototherapy). PLGA can encapsulate both a therapeutic drug and an imaging contrast agent (e.g., fluorescent dyes, MRI agents) to allow imaging-guided therapy. This enables tracking of nanoparticles’ biodistribution, accumulation in tumours, and therapeutic response [32]. For example, PEG-PLGA particles embedding Fe_3_O_4_ magnetic nanoparticles (for MR imaging) in the shell and Prussian blue (photothermal agent) inside, plus doxorubicin in the core, have been used for ultrasound/MR/photoacoustic imaging and combined chemo-photothermal therapy [91]. PLGA NPs have been developed that co-encapsulate chemotherapeutic drugs along with photosensitizers (or photothermal dyes), achieving chemophototherapy. For instance, carboplatin and indocyanine green (ICG) were co-loaded into PLGA NPs, enabling both photodynamic ROS generation and photothermal effects under NIR irradiation [92]. Some PLGA-based systems are engineered to respond to multiple triggers, e.g., pH, NIR light, or external magnetic fields, to release payloads in a controlled fashion. For instance, the particles mentioned above (Fe_3_O_4_ + Prussian blue + DOX) can release the drug faster under NIR irradiation (because heating raises polymer mobility) and under a magnetic field (which helps reposition the capsule) [91]. Such designs improve safety (minimise premature release), enhance targeting, and enable spatiotemporal control over therapy. By combining therapy and diagnostics, these PLGA NPs allow real-time monitoring of where the therapy goes, how much accumulates, and whether the treatment is effective. Co-delivering multiple functional agents (drug, immunomodulator, and photothermal agent) can yield synergistic therapeutic effects that are greater than the sum of individual modalities. External stimuli (light, magnetic field) or internal stimuli (pH, microenvironment) can be used to trigger release on demand, increasing local efficacy and reducing systemic toxicity. There are many advantages of multimodal delivery systems; however, designing NPs that stably encapsulate very different agents (a hydrophobic dye, a hydrophilic drug, and an immune agonist) in a controlled manner is nontrivial. Multi-cargo systems are often more difficult to scale for clinical translation. More components mean more potential for unexpected interactions, immunogenicity, or toxicity. Theranostic systems often face more regulatory hurdles because they combine diagnostic and therapeutic functions.

### 4.6. Case Studies of Co-Delivery Platforms

Many recent studies have demonstrated the potential of PLGA-based co-delivery systems across diverse biomedical applications. Recently, Mahanta et al. designed a nonviral delivery system capable of transporting both drugs and genes to the brain via a non-invasive intranasal route [93]. They developed mannose-coated mPEG-PLGA nanoparticles, utilising mannose as a brain-targeting ligand, to co-deliver cannabidiol (CBD) and a plasmid construct encoding the apolipoprotein E2 (ApoE2) isoform (pApoE2). The resulting CBD-loaded, mannose-coated nanoparticles exhibited an average diameter of 179.3 ± 4.57 nm and a zeta potential of 30.3 ± 6.45 mV. These coated nanoparticles provided sustained CBD release, with approximately 93% of the payload released over 30 days. Compared with free CBD, the nanoparticle formulation significantly reduced lipopolysaccharide- and amyloid β-induced inflammation in immortalised microglial cells. The study demonstrated that mannose-conjugated, chitosan-coated PLGA nanoparticles could function as a nonviral delivery platform for simultaneous drug and gene delivery to the brain via the intranasal route, offering a promising approach for Alzheimer’s disease management [93].

Cruz et al. developed CRISPR/Cas9-loaded PLGA nanoparticles (CRISPR/Cas9-PLGA-NPs) capable of efficiently encapsulating the Cas9 protein, single guide RNA (sgRNA), and a fluorescent probe [94]. The nanoparticles exhibited an initial burst release of Cas9 and sgRNA, followed by a sustained release profile. These CRISPR/Cas9-PLGA-NPs were effectively internalised and processed by human hematopoietic stem and progenitor cells (HSPCs) without causing cytotoxic effects. Following lysosomal escape, the nanoparticle-mediated delivery enabled gene editing at the γ-globin locus, leading to increased foetal haemoglobin (HbF) expression in primary erythroid cells. This CRISPR/Cas9-PLGA nanoparticle platform represents a promising nonviral approach for delivering CRISPR components to HSPCs and holds potential for in vivo therapies targeting hemoglobinopathies and other genetic disorders [94].

Encapsulating protein therapeutics in PLGA nanoparticles offers protection, sustained release, reduced side effects, and potential for combination therapy. To overcome the low throughput of conventional methods, Martins et al. used microfluidics for continuous nanoparticles production and co-delivery of two model proteins—BSA-FITC and BSA-TRITC. The process yielded uniform 100 nm NPs with 70% protein loading efficiency and a production rate of ~7 g/day, demonstrating scalability. The nanoparticles provided controlled protein release without structural damage and maintained >70% cell viability in macrophage-like cells. Cellular uptake of co-formulated proteins was 2× higher than free proteins and 4× higher than separate NP mixtures. This study establishes microfluidic PLGA NPs as an efficient platform for scalable protein co-delivery and future combination nanotherapies [95].

PLGA nanoparticles co-encapsulating doxorubicin and siRNA targeting multidrug resistance genes have shown synergistic tumour suppression while reducing systemic toxicity [96]. Similarly, dual-drug PLGA systems combining chemotherapeutics with anti-angiogenic agents enhanced tumour penetration and inhibited metastasis in murine models [97]. In case of formulating a drug delivery system as a PLGA shell and inorganic biomineral core, each of these components was carrying one drug form and controlling their release using a combination of polymer biodegradation and bio-mineral resorption. Particularly the coupling between biodegradation and bioresorption controlled the local increase in the acidity, preventing local inflammation and contributing to the drug release kinetics and ensuring the required active levels of the antibiotic, which locally prevented bacterial growth and ensured osteogenic differentiation and bone healing applicable for specific diseases as osteomyelitis [23,98]. Local drug delivery represents a promising strategy for breast cancer treatment by targeting the tumour site and reducing systemic toxicity. A pH-responsive silk fibroin film incorporating doxorubicin-loaded PLGA nanoparticles and simvastatin (SV/DOX PLGA/SF) was developed to prevent tumour recurrence. The optimised nanoparticles showed uniform morphology and controlled, pH-dependent release. Combined drug treatment demonstrated a synergistic effect, leading to enhanced tumour suppression and reduced recurrence in animal models. Histological analysis confirmed the therapeutic benefit of localised SF-based delivery [99].

Traditional vaccine adjuvants often fail to trigger both systemic and mucosal immunity, limiting early protection against pathogens. Yang et al. developed PLGA nanoparticle adjuvant (Res/RA QCS NPs) with a quaternized chitosan (QCS) coating for ovalbumen (OVA) delivery [100]. The nanoparticles co-encapsulate resveratrol and all-trans retinoic acid, achieving high antigen adsorption and enhanced antigen-presenting cell recruitment and lymph node targeting. In vitro, OVA-Res/RA QCS NPs promoted strong immune activation, efficient antigen uptake, dendritic cell maturation, cytokine release, and mucosal homing. In vivo, they induced robust serum IgG, T-cell proliferation, and intestinal IgA production, achieving simultaneous systemic and mucosal immune responses. This work offers a promising framework for next-generation adjuvants and intestinal immunotherapy strategies.

In infectious diseases, PLGA co-delivery of antibiotics and anti-inflammatory agents has improved bacterial clearance and mitigated tissue inflammation in wound infection models [101]. A hybrid PLGA system co-encapsulating poly(l-glutamic acid)-capped silver nanoparticles and ascorbic acid was developed to combine antimicrobial and antioxidant effects. The spherical particles exhibited high encapsulation efficiency, controlled release, and good biocompatibility in HepG2 cells. They effectively reduced intracellular ROS and demonstrated strong, sustained antimicrobial activity, highlighting their potential for infection treatment with simultaneous oxidative stress protection [102]. In case of formulating a drug delivery system as a PLGA shell and an inorganic biomineral core, each of these components was carrying one drug form and controlling their release using a combination of polymer biodegradation and bio-mineral resorption. Particularly, the coupling between biodegradation and bioresorption controlled the local increase in the acidity, preventing local inflammation and contributing to the drug release kinetics and ensuring the required active levels of the antibiotic, which locally prevented bacterial growth and ensured osteogenic differentiation and bone healing applicable for specific diseases as osteomyelitis [23].

For regenerative medicine, PLGA nanoparticles co-delivering growth factors (e.g., BMP-2) and angiogenic peptides demonstrated accelerated bone regeneration and improved vascularisation in preclinical studies [103]. A novel PLGA-based nanocarrier co-encapsulating quercetin and curcumin was developed for anti-osteoporotic therapy. The nanoparticles were uniform, stable, and cytocompatible, showing high encapsulation efficiency and sustained release. In vivo administration in ovariectomized rats improved bone mineral density and microarchitecture, effectively preventing trabecular bone loss. These results highlight the potential of flavonoid-loaded PLGA nanoparticles as a promising approach for osteoporosis treatment [104].

Multifunctional PLGA nanoparticles (~160 nm) were prepared by coupling PLGA with methoxy-PEG and chlorin e6 and incorporating Fe_3_O_4_ via a double-emulsion method. The Ce6 provided photodynamic and luminescent imaging capability, while Fe_3_O_4_ enabled MRI contrast. In vivo, these nanoparticles enhanced tumour imaging and achieved marked tumour regression under light irradiation, outperforming commercial contrast agents [105]. These examples highlight the versatility of co-delivery strategies in enhancing therapeutic efficacy, overcoming biological barriers, and achieving multifunctional outcomes tailored to specific disease contexts.

At the same time, several study-specific limitations surfaced that frame the translational gaps of these systems more clearly. For example, the CRISPR/Cas9 PLGA platform required careful optimisation to prevent loss of Cas9 and sgRNA activity during emulsification, since both components showed sensitivity to shear stress and organic solvents, which limited reproducibility across batches [94]. The microfluidics-based protein co-delivery system also faced operational challenges: channel clogging at higher protein concentrations, shifts in nanoparticle size distribution when flow rates were scaled up, and the need for frequent tuning of mixing ratios to maintain encapsulation efficiency [95]. These issues point to the practical barriers that still need attention when moving from proof-of-concept studies to robust, manufacturing-ready PLGA co-delivery platforms.

So, despite their therapeutic potential, PLGA-based co-delivery systems still face limitations that hinder their translational viability [106]. Manufacturing remains complex, as co-encapsulation of multiple cargo types, particularly combinations of small molecules and nucleic acids, requires precise control over formulation parameters, solvent systems, and mixing conditions. Scale-up is often challenging because many techniques, such as double emulsion or layer-by-layer assembly, may not be easily translated to industrial production. Additionally, high-purity reagents and rigorous control of sterile and endotoxin-free conditions are required to preserve the bioactivity of sensitive genetic payloads, increasing production time and cost. Batch-to-batch variability, especially in particle size, surface charge, and loading efficiency, can compromise reproducibility and regulatory approval. Finally, complex quality control and stability testing are needed to ensure the integrity of multicomponent formulations during storage and transport. These practical constraints highlight the need for improved, scalable, and cost-effective manufacturing strategies before PLGA-based multi-payload nanocarriers can be broadly implemented in clinical settings. Table 2 provides a comparative overview of various PLGA-based co-delivery formulations, highlighting their composition, fabrication approach, types of payloads, advantages, limitations, and representative recent references.

## 5. Mechanisms Governing Drug Release

### 5.1. Influence of Polymer Properties on PLGA Degradation

The composition of PLGA strongly affects how the polymer degrades, whether through bulk erosion, in which the entire matrix undergoes breakdown, or surface erosion, in which mass loss occurs mainly at the exterior. These pathways shape drug release kinetics and influence biocompatibility [111]. Higher glycolide content increases hydrophilicity and water uptake, which promotes faster bulk hydrolytic degradation, while higher lactide content slows the process [112]. Molecular weight also determines degradation rate. Low molecular weight PLGA breaks down more quickly, whereas high molecular weight polymers retain their structure for longer, delaying mass loss and extending release profiles [31]. End groups add another layer of control. Acid-terminated polymers often accelerate hydrolysis through autocatalysis, while ester-terminated variants degrade at a slower pace [113]. Because water usually penetrates PLGA faster than hydrolysis proceeds, bulk erosion is the predominant mechanism. Surface erosion appears only when the polymer structure or geometry restricts water diffusion [112]. By tuning lactide to glycolide ratios, molecular weights, and end groups, it is possible to design PLGA carriers with predictable degradation and release behaviour that meet biocompatibility requirements.

### 5.2. The Physical and Chemical Mechanisms Governing Drug Release

**Figure 4:**
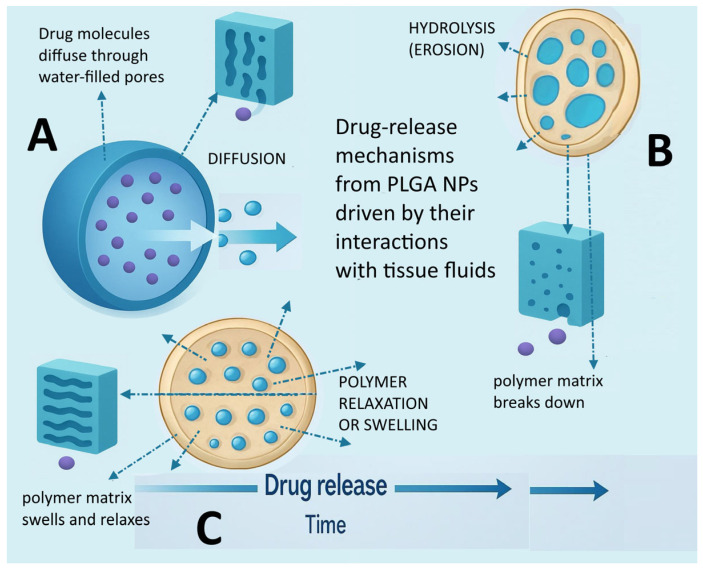
Illustration of the mechanisms governing drug release from PLGA nanoparticles driven by their interactions with tissue fluids: diffusion (**A**), hydrolysis-driven erosion (**B**) and polymer relaxation or swelling (**C**).

Drug release from PLGA systems arises from interconnected physical and chemical processes that evolve. Diffusion, polymer relaxation or swelling, and hydrolysis-driven erosion collectively govern release kinetics (Figure 4) [114]. Bulk erosion occurs when water enters the matrix faster than hydrolysis occurs and leads to interior degradation with increasing porosity and an initial burst release (Figure 4A), whereas surface erosion occurs when hydrolysis is faster than water penetration and results in layer-by-layer mass loss, more linear release, and gradual shrinkage (Figure 4B). Early in the process, drug molecules diffuse through water-filled pores or microchannels according to Fick’s law, which typically produces a release rate proportional to the square root of time. As the polymer swells and chain mobility increases, relaxation and rearrangement of chains expand free volume and enhance diffusion (Figure 4C). At later stages, hydrolysis of ester bonds increases porosity and weakens the matrix, which speeds drug release. These reactions generate acidic products that lower local pH and further promote degradation. In core shell structures with mineral cores, the acidic species may be buffered, moderating hydrolysis and slowing drug release [22]. As already mentioned above, hydrolytic degradation of PLGA generates acidic products (lactic and glycolic acids) that can locally lower pH within the particle, accelerating degradation and potentially destabilising pH-sensitive drugs. To counteract this, several formulation strategies have been employed. Incorporation of buffering agents, such as magnesium hydroxide or calcium carbonate, can neutralise acidic microenvironments [115]. Similarly, blending PLGA with other polymers (e.g., polyethyleneimine, chitosan) or hydrophilic excipients can moderate local pH and reduce autocatalysis. Inorganic cores or mineral additives within core–shell nanoparticles can also interact with acidic degradation products, slowing local acidification and stabilising encapsulated therapeutics. Such strategies are particularly important when co-delivering labile molecules, such as nucleic acids or proteins, ensuring sustained bioactivity and predictable release kinetics.

By adjusting polymer composition, morphology, and processing conditions, formulations can be designed with defined burst, lag, and sustained release phases tailored to therapeutic needs [116].

### 5.3. Relevant Mathematical Modelling Approaches

Mathematical and experimental modelling has become an important tool for understanding and optimising drug release from PLGA-based co-delivery systems [117]. Mechanistic models that include water entrance, hydrolytic cleavage, drug dissolution, diffusion, and matrix erosion can predict release profiles across a range of formulations [118]. Studies on PLGA microspheres highlight how autocatalysis and mass transfer limitations influence bulk erosion behaviour [119]. Semi-empirical models such as the Weibull and Korsmeyer–Peppas equations remain widely applied, and comparative analyses often show the Weibull model provides stronger fits for many PLGA nanoparticle systems [120]. More recently, data-driven strategies using machine learning and neural networks have gained attention. For instance, the DrugNet model applies multilayer perceptron algorithms to integrate polymer composition, drug properties, and release data, achieving lower mean square error and higher R^2^ values than conventional approaches [120,121]. Experimental studies involving co-formulated cargos manufactured using microfluidic PLGA systems further demonstrate how combining modelling with controlled production helps predict and validate co-release behaviour [95]. However, while the combination of mechanistic or data-driven modelling with microfluidic fabrication has proven highly effective for optimising PLGA-based co-delivery systems at the laboratory scale, translating this approach to industrial production presents several challenges [41]. Microfluidic techniques provide precise control over particle size, morphology, and payload distribution, enabling accurate validation of release models; however, scaling up microfluidic processes to produce large batches remains complex and often cost-prohibitive. High-purity reagents, stringent sterile and endotoxin-free conditions, and precise process control are required, increasing operational costs. Standardisation is also a concern: slight variations in flow rates, channel geometry, or polymer–solvent interactions can impact particle uniformity and release behaviour, making reproducibility and regulatory compliance more challenging at larger scales. Furthermore, integrating predictive modelling into high-throughput manufacturing requires robust process analytical technologies (PAT) and real-time monitoring systems to ensure that experimental parameters translate accurately to industrial settings. Despite these limitations, the combined use of modelling and microfluidics remains a powerful tool for rational design [41], enabling targeted optimisation of co-delivery nanocarriers prior to scale-up and helping to reduce development time and experimental waste. Together, these approaches support a rational and predictive design-for-release framework.

### 5.4. Programmed Release Strategies for Co-Delivery Systems

Achieving controlled sequential or simultaneous release in PLGA co-delivery systems is essential for synergistic therapeutic effects and reduced toxicity. Release kinetics can be tuned through polymer composition, such as adjusting the lactic to glycolic acid ratio or molecular weight to influence hydrophobicity and degradation rate. These adjustments allow systems to shift between rapid and sustained release profiles. It is important to note that, in addition to passive strategies that control release through polymer composition, molecular weight, and particle architecture, PLGA-based co-delivery systems can also achieve active, stimuli-responsive control. By responding to external triggers (e.g., temperature, light, magnetic fields) or microenvironmental cues (e.g., pH, redox potential, enzymatic activity), these systems enable site-specific and temporally precise release of therapeutic agents, complementing the passive, intrinsic tuning of release kinetics. Core–shell and multilayered nanoparticles can spatially separate cargos with different physicochemical properties, enabling programmed sequential release [122]. Processing factors such as solvent choice, emulsification energy, and surfactant concentration further refine encapsulation efficiency and diffusion rates, which directly affect release timing. Hybrid systems that combine PLGA with pH-, redox-, or enzyme-responsive materials introduce stimulus-triggered release in specific environments, including tumours and inflamed tissues [123]. By leveraging mechanistic insights into polymer behaviour, internal architecture, and fabrication parameters, next-generation PLGA-based co-delivery systems can be rationally designed to achieve precise, predictable, and stimuli-responsive release profiles. This mechanistic understanding also provides a foundation for subsequent surface modification and functionalization strategies, enabling targeted delivery and enhanced therapeutic efficacy.

## 6. Surface Modification and Functionalization

**Figure 5:**
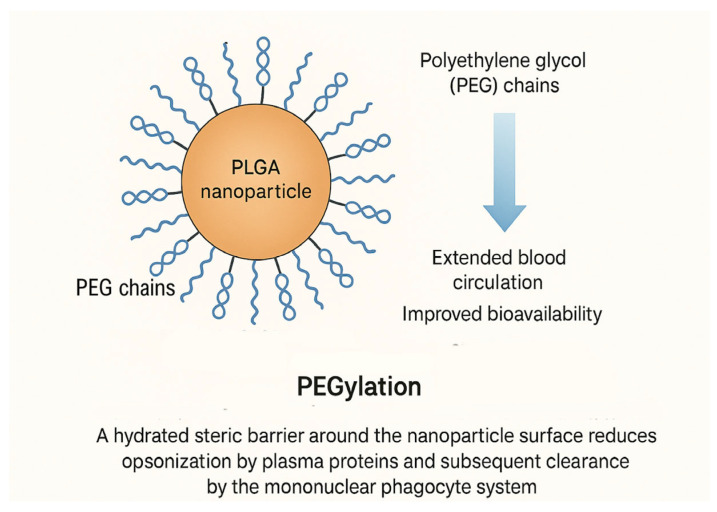
PEGylated PLGA nanoparticle.

PEGylation, the covalent attachment of polyethylene glycol (PEG) chains to the surface of PLGA nanoparticles, is one of the most effective strategies for enhancing stealth behaviour and prolonging systemic circulation. PEG forms a hydrated steric barrier around the nanoparticle surface that reduces opsonisation by plasma proteins and subsequent clearance by the mononuclear phagocyte system, thereby extending blood half-life and improving bioavailability (Figure 5) [124]. While PEGylation effectively improves circulation time, excessive PEG density can sterically hinder or partially mask surface-exposed targeting ligands such as antibodies, peptides, or aptamers, thereby reducing receptor binding and active cellular internalisation. Optimising PEG chain length and grafting density or using cleavable PEG coatings has therefore become essential to balance stealth properties with efficient ligand-mediated targeting [125,126].

The PEG chain length and density critically influence these effects: higher PEG coverage improves stealth properties but may reduce cellular uptake or drug loading efficiency [127]. In PLGA–PEG copolymers, PEG acts not only as a hydrophilic shield but also as a structural modifier that enhances colloidal stability and prevents aggregation in physiological conditions [97]. PEGylated PLGA nanoparticles have demonstrated prolonged plasma retention and improved therapeutic outcomes in cancer, inflammation, and brain-targeted delivery by evading immune recognition and enhancing passive tumour accumulation via the EPR effect [97]. However, recent studies also highlight emerging concerns regarding anti-PEG antibody formation and potential immune responses upon repeated administration. In addition to conventional PEGylation, which has historically served as the gold standard for reducing opsonisation and prolonging circulation, recent advances have introduced next-generation stealth strategies that overcome limitations such as anti-PEG immunity. Alternative hydrophilic coatings, including poly(zwitterions), poly(2-oxazoline)s, and polysarcosine, offer improved antifouling performance and reduced immunogenicity [128]. These coatings minimise protein adsorption by forming highly hydrated, charge-neutral surfaces that resist nonspecific interactions through strong ion–dipole or hydrogen-bond–mediated water structuring; unlike PEG, these materials do not trigger complement activation or anti-polymer antibody formation, offering superior long-term stealth performance and reduced immunogenicity [129,130]. A recent study using zwitterionic–liquid-coated nanoparticles derived from PLGA demonstrated that such coatings lead to low serum protein adsorption and limited hemolysis, indicating favourable biocompatibility and stealth behaviour compared to uncoated or PEGylated PLGA nanoparticles [131]. Ligand-based targeting represents a powerful strategy for enhancing the selectivity and efficiency of drug delivery systems by exploiting specific molecular interactions between targeting ligands and receptors overexpressed on diseased cells or tissues. Antibodies are among the most widely used ligands due to their high specificity and strong affinity for target antigens, enabling precise recognition of cancer or inflammatory cells and facilitating receptor-mediated endocytosis of therapeutic payloads [132]. Peptides, in contrast, offer advantages such as smaller size, lower immunogenicity, and easier chemical modification, making them attractive for targeting receptors like integrins (e.g., RGD motif) or growth factor receptors. Aptamers, synthetic single-stranded DNA or RNA oligonucleotides, have also emerged as versatile ligands with antibody-like binding affinity but superior stability and synthetic tunability [133]. By incorporating these ligands into nanocarriers or hydrogel matrices, ligand-based targeting enables site-specific accumulation, improved cellular uptake, and reduced systemic toxicity, thus significantly advancing the precision and efficacy of modern therapeutic delivery platforms. Peptide- or protein-based ligands, such as arginine-glycine-aspartic acid (RGD) peptides, transferrin, or antibody fragments, can be conjugated to the nanoparticle surface to promote receptor-mediated internalisation into target cells, significantly improving accumulation at disease sites and reducing off-target effects [134]. In parallel, coating PLGA nanoparticles with cell-derived membranes such as cancer cell membranes, erythrocyte membranes, or immune cell membranes provides biomimetic interfaces that confer homotypic targeting, prolonged circulation, and immune evasion, thereby enhancing delivery precision and therapeutic efficacy [135]. These functionalization strategies are increasingly integrated into co-delivery systems to ensure that both payloads reach the intended biological compartments with maximal efficiency. Cationic coatings such as chitosan and polyethyleneimine (PEI) play a crucial role in nucleic acid delivery by facilitating electrostatic complexation with negatively charged DNA or RNA molecules, protecting them from enzymatic degradation, and promoting efficient cellular uptake [136]. Chitosan, a naturally derived polysaccharide with primary amine groups, offers biocompatibility, biodegradability, and mucoadhesive properties that enhance transfection efficiency while minimising cytotoxicity [137]. Its protonable amino groups enable pH-responsive release within endosomal environments, aiding endosomal escape of nucleic acids into the cytoplasm. PEI, on the other hand, is a synthetic polymer with a high cationic charge density that condenses nucleic acids into compact nanoparticles through strong electrostatic interactions, exhibiting robust transfection efficiency in various cell types. However, its use is often limited by dose-dependent cytotoxicity, prompting strategies such as surface modification, PEGylation, or hybridisation with biopolymers like chitosan to balance transfection performance and biocompatibility. Overall, cationic coatings based on chitosan and PEI remain central to the design of non-viral vectors for safe and efficient nucleic acid delivery.

## 7. Clinical Translation and Regulatory Considerations

### 7.1. Clinical Translation

PLGA-based formulations have been extensively studied in clinical trials, demonstrating their versatility in drug delivery across various therapeutic areas. These biodegradable polymers are particularly valuable for developing long-acting injectable (LAI) formulations, which offer sustained release profiles for chronic conditions. For instance, PLGA microspheres have been utilised in LAI formulations for leuprolide acetate, a treatment for prostate cancer and endometriosis, allowing for extended dosing intervals and improved patient compliance [138,139]. PLGAs remain among the few biodegradable synthetic biomaterials currently used in FDA-approved parenteral long-acting release (LAR) products [140]. In the field of oncology, PLGA-based nanoparticles have been investigated for their potential to encapsulate chemotherapeutic agents, enhancing drug stability and targeting efficiency. These formulations aim to improve therapeutic outcomes while minimising systemic toxicity [141]. Furthermore, PLGA-based systems are being explored in the treatment of neurodegenerative diseases. For example, PLGA nanoparticles have been evaluated for delivering dopamine agonists and other agents to the brain, addressing challenges associated with blood–brain barrier penetration and providing sustained drug release [142]. These clinical investigations underscore the broad applicability and ongoing development of PLGA-based formulations in modern therapeutics.

A comparative look across therapeutic areas reveals that oncology and neurology stand out as particularly promising, and at the same time more challenging, fields for PLGA-based formulations. In oncology, PLGA excels because tumour tissues often benefit from sustained drug exposure, combination therapy, and protection of labile drugs from premature degradation, all of which align well with PLGA’s tunable release properties. However, tumour heterogeneity, variable EPR effects, and rapid drug efflux in resistant cancers demand sophisticated designs (targeting ligands, combination payloads), only some of which have been validated clinically. Neurological applications similarly benefit from PLGA’s capacity for sustained and targeted delivery but face additional hurdles such as blood–brain barrier penetration and the need for extremely tight control over particle size, surface chemistry, and release kinetics. Compared with more accessible areas like endocrinology or pain management, where PLGA depots are already routine, oncology and neurology therefore offer higher therapeutic impact but also require more complex engineering and regulatory evidence before broad clinical adoption becomes feasible.

Most recent clinical translations in the 2020–2025 window are dominated by long-acting depot/microsphere PLGA products (intra-articular and systemic LAIs) because the regulatory path for PLGA depots is relatively well established; newer hybrid co-delivery nanoparticles (lipid–PLGA for mRNA, inorganic-PLGA theranostics) are strongly represented in preclinical and early translational studies, but only a few have reached mid-stage clinical testing by 2024–2025. Recent clinical trials involving PLGA-based nanoparticles for co-delivery of multiple components have begun to appear primarily in immunotherapy and vaccine development. One representative example is an early-phase clinical study (NCT04751786) investigating PLGA nanoparticles co-encapsulating NY-ESO-1 peptide antigens together with an invariant NKT-cell agonist. This trial illustrates the translational feasibility of antigen–adjuvant co-delivery in a clinically regulated environment and reflects a broader trend toward immunomodulatory nanovaccines.

By contrast, most co-delivery platforms involving mRNA or other genetic payloads remain largely in preclinical or very early translational stages. The clinical landscape for nucleic-acid therapeutics is currently dominated by lipid nanoparticles (LNPs), while hybrid systems such as lipid–PLGA or inorganic–PLGA carriers are still undergoing optimisation for stability, manufacturability, and regulatory compliance before advancing to mid-stage clinical evaluation.

For comprehensive listings of PLGA products and trials, recent reviews summarising ClinicalTrials.gov entries can be consulted [143].

Although most PLGA-based co-delivery systems discussed in the literature involve parenteral applications, there is growing interest in translating co-loaded PLGA platforms to non-invasive routes. PLGA-based nanoparticles have also been explored for transmucosal, topical, and oral delivery, where they can enhance drug stability, improve mucosal permeability, and provide controlled local or systemic release, further demonstrating the versatility of these platforms. For example, PLGA NPs formulations (including conjugate-loaded and surface-modified systems) investigated for wound healing and topical delivery, often combining antimicrobial agents with growth factors or anti-inflammatory drugs to accelerate repair [144]. However, current reports of truly co-delivering PLGA systems (e.g., drug + gene) via oral or topical administration remain limited, highlighting a translational gap and an opportunity for future research in non-parenteral co-delivery technologies.

### 7.2. Regulatory Status of PLGA and Co-Loaded Nanocarriers (FDA, EMA)

Poly(lactic-co-glycolic acid) (PLGA) has been extensively studied and utilised in biomedical applications, leading to its approval by both the U.S. Food and Drug Administration (FDA) and the European Medicines Agency (EMA) for various medical uses [139]. These include drug delivery systems, vaccines, and tissue engineering applications. Although not at the nanoscale, more than 60 PLGA-based drug products with diverse properties are already marketed. Well-known examples include microparticle depot formulations such as: Decapeptyl^®^ (the first marketed drug formulation containing triptorelin), Lupron Depot^®^ (leuprolide acetate), Nutropin Depot^®^ (somatropin), Suprecur^®^ MP (buserelin acetate), Sandostatin^®^ LAR Depot (octreotide acetate), Somatuline^®^ LA (lanreotide acetate), Trelstar™ Depot (triptorelin pamoate), Vivitrol^®^ (naltrexone) and Risperdal^®^ Consta™ (risperidone). In addition, PLGA-based implants (e.g., Zoladex^®^, Ozurdex^®^, Profact^®^ Depot, Durysta™, etc., based on goserelin acetate, dexamethasone, buserelin and bimatoprost, respectively) and even in situ forming implants based on Atrigel^®^ system, i.e., Eligard^®^ (leuprolide acetate), are available [48,140]. This strong market presence underscores the potential of PLGA-based systems for future nanomedicine development. Table 3 summarises selected approved PLGA-based long-acting formulations, including their active ingredient, indication, administration route, and year of approval.

PLGA’s approval stems from its biocompatibility, biodegradability, and the ability to tailor its degradation rates to suit specific therapeutic needs [17,145]. Regarding co-loaded nanocarriers, which combine multiple therapeutic agents within a single delivery system, regulatory agencies evaluate each formulation on a case-by-case basis. The FDA and EMA assess these systems through rigorous preclinical and clinical studies to ensure safety, efficacy, and quality. While PLGA-based co-loaded nanocarriers have shown promise in research and early clinical trials, their regulatory approval depends on comprehensive data demonstrating their performance and safety profiles [139].

**Table 3:** Approved PLGA-based long-acting formulations.

Brand Name	Active Ingredient	Indication	Method	Route of Administration	Year of Approval	Refs.
Decapeptyl^®^	Triptorelin pamoate	Inhibition of gonadotropin secretion (Prostate cancer)	NA	Intramuscular injection	1986 (EU)	[77,146]
Lupron Depot^®^	Leuprolide acetate	Advanced prostate cancer, endometriosis, fibroid	Water-in-oil emulsification	Intramuscular, monthly	1989 (FDA), 1995, 1997, 2011	[147]
Zoladex^®^	Goserelin acetate	Advanced breast cancer in pre-and perimenopausal women, endometriosis, prostate cancer	Hot melt extrusion	Subcutaneous implant	~1989	[148]
Sandostatin^®^ LAR Depot	Octreotide acetate	Acromegaly	Emulsion solvent evaporation	Intramuscular microspheres	1998	[149]
Nutropin Depot^®^	Somatropin	Growth hormone deficiency	Spray drying	Subcutaneous injection	1999	[150]
Eligard^®^	Leuprolide acetate (in situ PLGA implant)	Advanced prostate cancer	PLGA dissolved in a biocompatible solvent such as N-methyl-2-pyrrolidone	Subcutaneous injection	2002	[146,151]
Risperdal^®^ Consta™	Risperidone	Schizophrenia, bipolar disorder	Emulsion solvent evaporation	Intramuscular, every 2 weeks	2003	[149]
Vivitrol^®^	Naltrexone	Alcohol dependence, opioid dependence	Emulsion solvent evaporation	Long-acting intramuscular injection	2006	[146]
Somatuline^®^ LA	Lanreotide acetate	Acromegaly, carcinoid syndrome	Spray drying	Intramuscular PLGA microparticles	2007	[147]
Triptodur™	Triptorelin pamoate	Central precocious puberty	Oil-in-water emulsification/	Intramuscular injection	2017	[77]
Perseris™	Risperidone	Adult schizophrenia	NA	Subcutaneous injection	2018	[151]
Fensolvi^®^	Leuprolide acetate	Central precocious puberty	NA	Subcutaneous injection	2020	[146]

Scaling up PLGA-based and co-loaded nanocarrier systems presents several critical challenges, particularly in maintaining reproducibility and dual-payload stability [48]. Achieving consistent particle size, morphology, and drug encapsulation efficiency at larger production scales is difficult due to the sensitivity of nanoparticle formation processes to parameters such as solvent choice, mixing rates, and polymer concentration. Reproducibility is further complicated when co-loading two therapeutics with distinct physicochemical properties, as differences in solubility, stability, or degradation kinetics can lead to uneven distribution, premature release, or reduced bioactivity [2]. Ensuring dual-payload stability also requires careful optimisation of polymer composition, surface modifications, and formulation conditions to prevent chemical interactions, aggregation, or differential release during storage and in physiological environments.

Addressing these challenges is essential for translating PLGA-based co-delivery platforms from laboratory-scale research to clinically viable, reproducible, and safe therapeutic products. Good Manufacturing Practice (GMP) manufacturing and translational research offer significant opportunities for advancing PLGA-based and co-loaded nanocarrier systems from bench to bedside [48]. Standardising formulation protocols under GMP conditions ensures reproducibility, sterility, and regulatory compliance, which are critical for clinical translation and large-scale production of nanomedicines. The modularity of PLGA platforms allows integration of multiple therapeutic agents, targeting ligands, or imaging components, facilitating the development of multifunctional systems suitable for personalised medicine applications. Translational research efforts, including preclinical in vitro and in vivo studies, enable optimisation of pharmacokinetics, biodistribution, and safety profiles, bridging the gap between laboratory findings and clinical implementation.

Moreover, advances in microfluidics, scalable nanoprecipitation, and automated encapsulation techniques are facilitating GMP-compliant production while maintaining particle uniformity and payload stability, thereby accelerating the path toward regulatory approval and real-world therapeutic applications [152].

Looking forward, several technological advances may accelerate the development and regulatory approval of co-loaded PLGA nanomedicines. Microfluidic platforms enable highly reproducible and scalable nanoparticle fabrication, while AI-driven formulation optimisation can predict release kinetics and improve co-loading efficiency. In-line monitoring and real-time quality control during manufacturing further ensure batch consistency and regulatory compliance, collectively supporting more rapid clinical translation of complex PLGA-based delivery systems.

## 8. Challenges and Future Perspectives

Despite the promising potential of co-loaded PLGA nanocarriers, practical barriers remain that may limit clinical translation. Challenges include ensuring long-term stability of diverse payloads, preventing aggregation or premature release, minimising immunogenicity, and achieving consistent performance across batches under GMP-compliant manufacturing. Variability in polymer properties, solvent residues, or encapsulation efficiency can compromise reproducibility, while complex dual- or multi-drug formulations may introduce unforeseen chemical interactions. Addressing these issues requires rigorous preclinical evaluation, robust formulation optimisation, and advanced manufacturing strategies to ensure safety, efficacy, and regulatory compliance in real-world applications. Co-delivery strategies, while offering the potential for synergistic therapeutic effects, face notable limitations related to payload compatibility and release synchronisation. Different drugs or biomolecules often possess distinct physicochemical properties, such as solubility, stability, and degradation kinetics, which can lead to interactions that compromise encapsulation efficiency or chemical integrity [24,153]. This is particularly evident in combinations such as hydrophilic and hydrophobic drugs, or chemotherapeutics co-delivered with nucleic acids (siRNA or mRNA), where differences in solubility and susceptibility to degradation complicate formulation. Achieving synchronised release is particularly challenging, as variations in polymer degradation, diffusion rates, or environmental responsiveness can result in premature or delayed delivery of one component relative to the other, potentially reducing efficacy or increasing toxicity [154]. Additionally, co-loading may influence nanoparticle stability, aggregation tendencies, and immune recognition, further complicating the design of safe and effective dual-drug carriers. Addressing these limitations requires careful selection of compatible payloads, tailored polymer matrices, and stimuli-responsive or compartmentalised architectures to achieve controlled, coordinated release and maximise therapeutic outcomes. The integration of machine learning (ML) and computational modelling into nanocarrier formulation has emerged as a powerful approach for optimising design parameters and predicting therapeutic performance [155]. ML algorithms can analyse large datasets from experimental studies to identify correlations between formulation variables, such as polymer composition, particle size, surface charge, and drug loading, and key outcomes like encapsulation efficiency, release kinetics, and cytotoxicity. Computational modelling, including molecular dynamics and finite element simulations, enables the prediction of nanoparticle behaviour under physiological conditions, guiding the rational design of carriers for enhanced stability, target specificity, and payload release synchronisation. Together, these approaches accelerate formulation optimisation, reduce experimental costs, and improve reproducibility, providing a data-driven framework for designing next-generation PLGA-based and co-loaded delivery systems with precise, predictable performance.

PLGA-based and co-loaded nanocarrier systems hold significant potential for personalised medicine through their modular and tunable design, which allows therapies to be tailored to individual patient profiles. By adjusting polymer composition, particle size, surface functionalization, and payload combinations, these platforms can be optimised for specific pharmacokinetics, target receptor expression, and disease microenvironments, enabling precise, patient-specific therapeutic delivery [156]. Co-delivery capabilities further facilitate the simultaneous administration of synergistic drugs, nucleic acids, or imaging agents, supporting combination therapies that address heterogeneity in tumour biology or chronic disease responses [157,158]. Additionally, the modularity of these systems allows integration with targeting ligands, stimuli-responsive elements, or imaging modalities, enhancing selectivity, controlled release, and real-time monitoring of therapeutic outcomes [159]. Collectively, this design flexibility positions PLGA-based nanocarriers as promising tools for advancing personalised and precision medicine, offering adaptable platforms for patient-specific treatments. Next-generation PLGA-based systems are poised to expand beyond conventional drug delivery toward advanced applications such as biodegradable implants and smart, stimuli-responsive nanoparticles. Biodegradable PLGA implants can provide long-term, localised therapy with minimal intervention, offering sustained release for chronic conditions, postoperative care, or tissue regeneration while gradually degrading into biocompatible metabolites [160]. Smart PLGA nanoparticles, engineered with pH-, temperature-, or redox-responsive functionalities, enable on-demand, site-specific release of therapeutics in response to pathological microenvironments, enhancing efficacy while reducing off-target toxicity. Integration with imaging agents, targeting ligands, or co-loaded therapeutics further allows multifunctional platforms for theranostics and personalised medicine applications [161]. Advances in scalable, GMP-compliant manufacturing and computational formulation optimisation are expected to accelerate translation of these next-generation PLGA systems into clinical practice, bridging the gap between laboratory innovation and patient-specific therapy [162]. Nevertheless, it must be noted that from an ethical and regulatory standpoint, the translation of personalised PLGA-based nanomedicines also raises important challenges. Ensuring traceability, standardisation, and batch-to-batch reproducibility of individualised formulations under GMP is nontrivial, especially when small-scale, patient-specific batches are manufactured. Regulators (e.g., FDA, EMA) currently lack fully harmonised frameworks for nanomedicines, and debates continue about how to control critical quality attributes (CQAs) and the safety of flexible, adaptive therapeutics. Emerging regulatory efforts emphasise risk-based approaches and Quality by Design (QbD) paradigms to manage complexity and variability in nano therapeutic development [163]. Moreover, ethical concerns around data privacy, patient consent, and algorithmic transparency must be addressed for personalised nanomedicine. As AI and ML tools are increasingly used for patient stratification and formulation optimisation, issues such as version control of predictive models, “locking” algorithm configurations, and long-term data ownership become relevant [164]. Regulatory authorities worldwide have undertaken scientific studies to address the challenges posed by emerging technologies, including nanotechnology. While the international academic community has yet to reach a consensus on the safety of nanomaterials, regulatory bodies uniformly emphasise the importance of thoroughly assessing and researching their safety in drugs and medical devices before market approval. In particular, long-term monitoring of medical nanomaterial implants is considered essential [165].

## 9. Conclusions

PLGA-based co-delivery nanocarriers have witnessed significant advances in recent years, combining the biocompatibility and tunable degradation of PLGA with the ability to simultaneously deliver multiple therapeutic agents. Key developments include the integration of targeting ligands, stimuli-responsive elements, and inorganic or polymeric hybrid components, which have enhanced site-specific delivery, controlled release kinetics, and multifunctional capabilities for imaging and therapy. Progress in formulation strategies, such as co-encapsulation of hydrophilic and hydrophobic drugs, modular polymer–polymer or lipid–polymer hybrids, and computationally guided design, has improved payload stability, reproducibility, and therapeutic efficacy. Future directions focus on personalised medicine applications, GMP-compliant scalable production, and smart, stimuli-responsive carriers capable of on-demand drug release, theranostic integration, and patient-specific therapy. Overall, the convergence of material innovation, computational modelling, and translational research positions PLGA-based co-delivery nanocarriers as a versatile platform for next-generation precision therapeutics. However, despite significant progress, several challenges remain that may hinder their clinical translation. Key bottlenecks include the absence of clear regulatory guidelines for co-loaded formulations, ensuring long-term stability of diverse payloads within the polymer matrix and achieving reproducibility and scalability under GMP-compliant manufacturing conditions. Overcoming these hurdles will require coordinated efforts in robust formulation optimisation, in-line process monitoring, and standardised quality control frameworks, ultimately enabling the safe and effective translation of these promising systems into clinical applications.

## Figures and Tables

**Table 1:** Fabrication strategies for PLGA-based nanoformulations.

Fabrication Strategy	Brief Description	Advantages	Limitations	Recent Reference(s)
Single Emulsion (Oil-in-Water, O/W)	PLGA and hydrophobic drug dissolved in an organic solvent → emulsified into aqueous surfactant phase → solvent evaporation to form nanoparticles.	Widely used for hydrophobic drugs; relatively simple.	Less suitable for hydrophilic drugs; possible residual solvent; broad size distribution.	Description of PLGA NP methods [53].
Double Emulsion (Water-in-Oil-in-Water, W/O/W)	Aqueous solution of hydrophilic cargo emulsified in organic PLGA solution, then re-emulsified in aqueous phase, followed by solvent evaporation.	Better for hydrophilic biomolecules (e.g., proteins, nucleic acids).	More complex, possible instability or loss of cargo; higher PDI.	Example: BMP-2 loaded PLGA NPs via W/O/W [54].
Nanoprecipitation (Solvent Displacement)	PLGA and drug in a water-miscible organic solvent are rapidly mixed into the aqueous phase → polymer precipitates forming nanoparticles.	Simpler process; good for hydrophobic drugs; potential for relatively small particles.	Control of mixing is critical; batch-to-batch reproducibility can suffer; less suitable for large biomolecules.	Enhanced nanoprecipitation method (2025) [55].
Microfluidics-Assisted Production	Use of microfluidic mixers or microreactors to precisely control mixing of polymer/drug and aqueous phases, enabling reproducible and uniform nanoparticle formation.	Excellent control of size, PDI; better reproducibility; scalable potential.	Requires specialised equipment; scaling up may require parallelisation; cost may be higher.	Modular microfluidic system for PLGA NP encapsulating proteins [56]. Also, an ultrasonic microreactor for PLGA NPs [57].
Spray Drying/Spray-Freeze Drying	Suspension of PLGA nanoparticles or microparticles is sprayed through hot air or frozen, then dried to form dry powder formulations.	Good for dry powder products, long-acting systems, and inhalable forms.	Heat or stress may degrade sensitive cargo; particle size control may be less precise than microfluidic methods.	Discussed in the implant/fabrication review context [58].
Electrospraying/Electrospray	Polymer/drug solution is ejected under a high electric field to form fine droplets, which solidify into particles.	Good for precise size control, high encapsulation potential, and suitable for sensitive cargo.	Lower throughput; technical setup complex; fewer examples in PLGA drug delivery compared to emulsion/nanoprecipitation.	Emerging method for polymeric particle synthesis (polymeric particle optimisation) [59].

**Table 2:** Comparison of co-delivery formulations.

Composition	Typical Structure/How It Is Made	Payload Types Suited	Key Advantages	Key Limitations/Risks	Representative Recent Refs
Lipid–PLGA core–shell	PLGA core (drug) with lipid shell (adsorbed/anchored nucleic acid or hydrophilic cargo); made by single-step nanoprecipitation + lipid coating or double emulsion + lipid assembly	Hydrophobic small molecules in core; mRNA/siRNA, proteins or adjuvants at/within shell	Spatial segregation for incompatible cargos; improved colloidal stability and membrane-mimetic interactions (better cell uptake)	Potential complexity in scale-up; shell detachment in vivo; stability of nucleic acids on/near surface.	[107,108]
Compartmentalised/multi-core (double emulsion, multicore)	Multiple aqueous/oil compartments (w/o/w) or multi-core droplets created by double emulsion or multi-phase nanoprecipitation	Hydrophilic (proteins, nucleic acids) + hydrophobic drugs simultaneously	Good separation of chemically incompatible payloads; tunable sequential release	Emulsion complexity; lower encapsulation efficiency for some cargos; reproducibility at scale.	[53,109]
Polymer–polymer blends/block co-polymers (PLGA–PEG, PLGA–PCL)	Physical blends or block copolymers formed during nanoprecipitation or solvent evaporation	Hydrophobic drugs, some proteins (with stabilisation)	Tuning of degradation and release kinetics; PEG improves stealth; simpler fabrication than multi-compartment systems	Phase separation risks; balancing hydrophilicity/hydrophobicity for dual payloads can be tricky.	[110]
Inorganic–PLGA hybrids (magnetic, gold, QDs, hydroxyapatite)	Inorganic core or embedded particles within the PLGA matrix, formed by co-encapsulation or surface adsorption	Imaging agents (iron oxide, QD), photothermal agents + chemotherapeutics, mineral–bonded fluorescent dye	Adds imaging/theranostic functionality; allows guided delivery or PTT/PDT, detection of degradation process	Added regulatory/toxicity burden for inorganic material; possible altered degradation and clearance.	[23,32]
